# Une pustulose palmoplantaire

**DOI:** 10.11604/pamj.2014.19.225.5486

**Published:** 2014-10-29

**Authors:** Faida Ajili, Imen Gharsallah

**Affiliations:** 1 Service de Médecine Interne, Hôpital Militaire de Tunis, 1008 Montfleury, Tunisie

**Keywords:** syndrome de Sapho, pustulose cutanée, scintigraphie osseuse, Sapho Syndrome, pustular rash, bone scintigraphy

## Image en medicine

Le syndrome SAPHO (synovite, acné, pustulose, hyperostose, osteite) est une entité rare initialement décrite par CHAMOT en 1987. Elle désigne l'association d'un ensemble hétérogène de manifestations cutanées et ostéoarticulaires ayant pour dénominateur commun un processus inflammatoire aseptique. La pustulose serait plus fréquente chez la femme et l'acné chez l'homme. Nous décrivons le cas d'un homme âgé de 31 ans, hospitalisé pour une pustulose palmoplantaire (A, B) évoluant depuis 3 semaines. Par ailleurs, il rapportait la notion de douleurs intermittentes de la paroi thoracique antérieure prédominant en regard de l'articulation sternoclaviculairedroite qui seraient apparues depuis deux mois et des douleurs lombosacrées d'allure inflammatoire. A l'examen, la pression de la paroi thoracique antérieure en regard du sternum et des premiers arcs costaux était douloureuse avec une tuméfaction et des signes inflammatoires locaux. La mobilisation des sacro-iliaques était douloureuse. La radiographie du bassin mettait en évidence une sacro-iléite débutante avec un pseudo élargissement de l'interligne articulaire et une irrégularité des berges iliaques. La radiographie du sternum et l’échographie des articulations sternoclaviculaire étaient sans anomalies. En revanche, la scintigraphie au technétium 99 concluait à une hyperfixation diffuse du sternum avec un foyer relativement intense de l'articulation sternoclaviculaire droite et deux petits foyers d'hyperfixations des articulations sacro-iliaques (C, D). Ainsi le diagnostic de SAPHO a été retenu devant les antécédents d'acné sévère, la pustulose palmoplantaire, les douleurs de la paroi thoracique antérieure et l'hyperfixation scintigraphique du squelette thoracique. Le patient est alors mis sous méthotrexate à la dose de 10 mg/semaine avec une régression quasi complète des lésions cutanées et une disparition des douleurs et de l'arthrite sternoclaviculaire.

**Figure 1:**
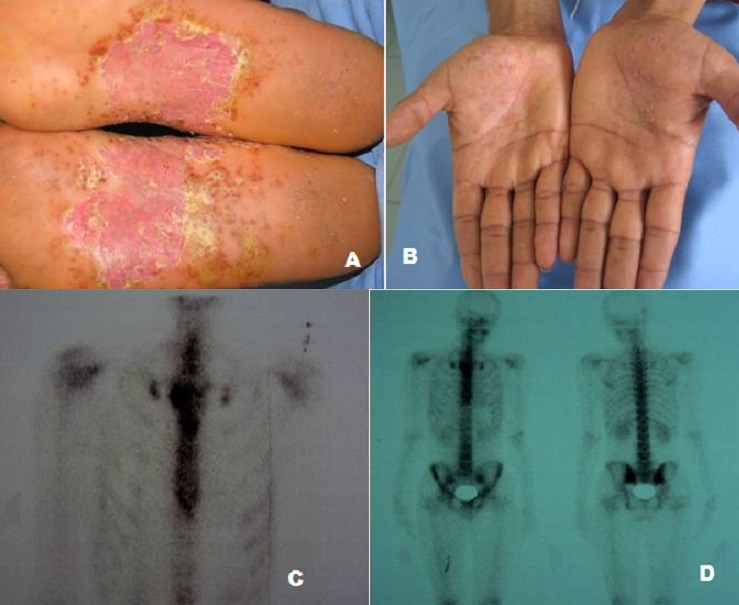
(A,B): pustulose palmoplantaire; (C): hyperfixation au niveau du sternum; (D): hyperfixation des articulations sacroiliaques

